# A pilot study of IL-1 inhibition by anakinra in acute gout

**DOI:** 10.1186/ar2143

**Published:** 2007-03-12

**Authors:** Alexander So, Thibaut De Smedt, Sylvie Revaz, Jürg Tschopp

**Affiliations:** 1 Service of Rhumatologie, Department of Medicine, Centre Hospitalier Universtaire Vaudois and University of Lausanne, 1011 Lausanne, Switzerland; 2 Apoxis SA, Avenue de Sévelin 18-20, 1004 Lausanne, Switzerland; 3 Institute of Biochemistry, University of Lausanne, chemin de Boveresses 155, 1066 Epalinges, Switzerland

## Abstract

Monosodium urate crystals stimulate monocytes and macrophages to release IL-1β through the NALP3 component of the inflammasome. The effectiveness of IL-1 inhibition in hereditary autoinflammatory syndromes with mutations in the NALP3 protein suggested that IL-1 inhibition might also be effective in relieving the inflammatory manifestations of acute gout. The effectiveness of IL-1 inhibition was first evaluated in a mouse model of monosodium urate crystal-induced inflammation. IL-1 inhibition prevented peritoneal neutrophil accumulation but TNF blockade had no effect. Based on these findings, we performed a pilot, open-labeled study (trial registration number ISRCTN10862635) in 10 patients with gout who could not tolerate or had failed standard antiinflammatory therapies. All patients received 100 mg anakinra daily for 3 days. All 10 patients with acute gout responded rapidly to anakinra. No adverse effects were observed. IL-1 blockade appears to be an effective therapy for acute gouty arthritis. The clinical findings need to be confirmed in a controlled study.

## Introduction

Acute gout is a common cause of arthritis, affecting approximately 1% of the adult population, and epidemiological evidence suggests that its prevalence is increasing [1]. Current treatments during an acute attack include nonsteroidal antiinflammatory drugs (NSAIDs), colchicine and corticosteroids. Although these agents are generally effective, they also present significant risks in patients who have pre-existing renal, cardiovascular and gastrointestinal diseases.

Gouty inflammation is due to monosodium urate (MSU) crystal-induced release of proinflammatory cytokines from leukocytes. Among the many cytokines implicated [2,3], IL-1 may have a special role in the inflammatory network, as MSU crystals stimulate IL-1 release by monocytes and synovial mononuclear cells [4]. The MSU crystals trigger IL-1 release through innate immune pathways, which include TLR-2 and TLR-4, found on the surface of monocytes and macrophages, as well as the 'inflammasome' complex that leads to IL-1β activation [5,6]. The inflammasome acts as an intracellular sensor of inflammatory stimuli and regulates the activation of caspase-1. On assembly of the inflammasome, which consists of a member of the nucleotide-binding oligomerization domain-leucine rich repeat protein family (such as NALP1, NALP2, NALP3, or IPAF), the adaptor protein ASC and caspase-1 [7], caspase-1 becomes active and cleaves pro-IL-1β to release the mature p17 form of IL-1β. Activators of the NALP3 inflammasome include ATP, the microbial cell-wall component muramyl dipeptide and bacterial RNAs [8-10]. MSU and calcium pyrophosphate dihydrate crystals directly activate the inflammasome via NALP3 in monocytes or macrophages to release active IL-1β and cause neutrophil influx into the peritoneal space when administered by intraperitoneal injection. These responses were abrogated in ASC^-/- ^or caspase-1^-/- ^mice [6].

Spontaneous activation of the NALP3 inflammasome due to mutations in the *NALP3 *gene has been implicated in hereditary autoinflammatory syndromes such as Muckle–Wells syndrome and chronic infantile neurologic cutaneous articular [11]. Affected patients respond dramatically to IL-1 inhibition [12,13], suggesting that IL-1β plays a crucial role in the pathogenesis of inflammation in these conditions. Based on these findings, we questioned whether IL-1 inhibition may also have a beneficial effect in gouty inflammation.

As treatment with drugs currently used in acute gout is not always well tolerated or is contraindicated due to coexistent medical problems, we investigated the validity of IL-1 blockade as therapy in acute gout. We first analyzed the effects of IL-1 inhibition using the mouse peritoneal model of MSU-induced inflammation and then assessed the effects of anakinra in patients with acute gout who presented contraindications or were refractory to standard treatment in an open study.

## Methods

### Reagents

MSU crystals were prepared based on the method described previously [14]. Briefly, 1.68 g uric acid was dissolved in 500 ml of 0.01 M NaOH and heated to 70°C. NaOH was added as required to maintain the pH between 7.1 and 7.2, and the solution was filtered and incubated at room temperature with little stirring slowly and continuously 24 hours.

### Animal studies

BALB/C mice were treated intraperitoneally with PBS or 0.5 mg MSU crystals in 0.5 ml sterile PBS. Some mice were injected intraperitoneally with 200 μg anti-IL-1RI mAb (clone 35F5; BD Pharmingen, San Jose, CA, USA) or with 200 μg anti-TNF mAb (clone TN3-19.12; BD Pharmingen) or with 200 μg anakinra (Kineret; Amgen, Thousand Oaks, CA, USA) at the time of MSU injection. Mice were euthanized after 6 hours by CO_2 _exposure and the peritoneal cavities were washed with 10 ml cold PBS. The lavage fluids were analyzed for neutrophil recruitment by fluorescence-activated cell sorting using the neutrophil markers Ly-6G and CD11b (BD Pharmingen). Five mice per group were used for study.

### Human studies

The diagnosis of gout arthritis was based on clinical and laboratory features. All patients fulfilled the American College of Rheumatology criteria for acute gouty arthritis [15], but all had a long previous history of either recurrent gouty attacks or tophaceous gout. Patients were treated with anakinra on an open-label basis. All subjects had either failed conventional treatment with NSAIDs, colchicine or corticosteroids for at least 48 hours or had developed significant side-effects on these drugs in the past. Patients with an active untreated infection, with uncontrolled diabetes, with uncontrolled heart or respiratory failure or with chronic renal failure with a creatinine clearance <30 ml/min were not eligible. Joint infection was excluded by prior bacterial culture in all cases that underwent joint aspiration.

All patients consented to receive anakinra. Treatment was administered daily at a dose of 100 mg subcutaneously for 3 days. On starting anakinra, the NSAID or colchicine therapy was discontinued. Patients who were already on low-dose corticosteroids continued their treatment at the same dose. Clinical efficacy was assessed by clinical examination of the swollen and tender joint count as well as the patient's evaluation of the efficacy of treatment in terms of pain reduction after 3 days of therapy in comparison with their symptoms before treatment. All patients were followed up for >1 month. During follow-up, patients were evaluated for side-effects related to the treatment and for joint symptoms related to gout. All patients were followed by physicians responsible for this study (AS and SR). The study protocol was approved by the local institution's ethics committee and has the ISRTCN trial registration number 10862635.

## Results

### MSU-induced peritoneal inflammation and its inhibition by anakinra or anti-IL-1RI mAb

**Figure 1:**
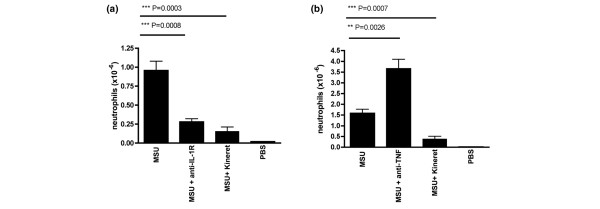
Inhibition of monosodium urate-induced peritoneal neutrophil influx by anti-IL-1 treatment. **(a) **BALB/C mice were injected intraperitoneally with 0.5 mg monosodium urate (MSU) crystals together with PBS or anti-IL-1RI mAb (200 μg) or anakinra (200 μg). **(b) **BALB/C mice were injected intraperitoneally with 0.5 mg MSU crystals together with PBS or anti-TNF mAb (200 μg) or anakinra (200 μg). Neutrophil influx in the peritoneum was quantified 6 hours later. Values are the mean ± standard error of the mean of five mice per group. An unpaired Student's *t *test was used to calculate the *P *value.

To study the inflammatory response to MSU crystals *in vivo*, we used a well-described model of neutrophil infiltration in the peritoneal cavity subsequent to MSU crystal administration. In previous experiments we determined that intraperitoneal administration of MSU crystals induced a dose-dependent neutrophil accumulation at the site of crystal deposition, with a plateau effect observed at 500 μg (data not shown), and this dose was therefore used in subsequent experiments. We went on to determine whether IL-1 blockade reduced the inflammatory response in the same model. Coadministration of MSU crystals with two different IL-1 inhibitors (anti-IL-1RI mAb or IL-1R antagonist (anakinra)) had similar and marked inhibitory effects on neutrophil recruitment (Figure 1a). This effect was not observed when anti-TNF blocking mAb was administered in the same fashion as the IL-1 inhibitors (Figure 1b). The differences were statistically significant in comparison with the positive MSU control.

The same dose of TNF inhibitor was effective in suppressing liver inflammation induced by concavalin A administration, a model of hepatitis dependent on TNF production (data not shown).

### Effects of anakinra in gout patients

The effectiveness of IL-1 inhibition in suppressing the symptoms of acute gout was assessed in 10 patients who could not tolerate or did not respond to standard antiinflammatory treatments. A summary of the medical histories is presented in Table 1. All patients responded rapidly to the drug, with the most rapid onset observed within 24 hours. In all patients, subjective symptoms of gout were greatly relieved by 48 hours after the first injection. No side-effects were observed during the study period. Clinical examination of affected joints showed complete resolution of signs of arthritis in 9/10 patients on day 3 after initiation of treatment.

**Table 1:** Clinical summary of the 10 patients studied and their response to treatment

Patient	Clinical presentation	Affected joints	Serum uric acid (normal range, 160–390 μmol/l)	Serum creatinine (normal range, 44–80 μmol/l)	Hypouricemic treatment	Effect of anakinra (hours)	Patient assessment of improvement in pain (%)
Case 1 (female, 72 years old)	Chronic tophaceous gout, renal stones	Fingers, toes	637	79	Uricase	36	70
Case 2 (male, 70 years old)	Chronic tophaceous gout	Ankle, toes	564	202	Allopurinol	24	90
Case 3 (male, 72 years old)	Acute gout	Knee, ankle, foot	482	121	Allopurinol	24	90
Case 4 (male, 51 years old)	Acute gout	Ankle, toe	396	84	Allopurinol	24	100
Case 5 (male, 40 years old)	Acute gout	Ankle, toe	322	113	Allopurinol	36	100
Case 6 (female, 72 years old)	Acute gout	Feet, toe	572	72	None	36	80
Case 7 (male, 76 years old)	Acute gout	Ankle, foot	338	79	None	36	100
Case 8 (male, 70 years old)	Acute gout	Wrist, elbow, hand	779	406	None	48	50
Case 9 (male, 53 years old)	Chronic tophaceous gout	Elbow, finger, foot, ankle	660	84	Allopurinol	48	50
Case 10 (male, 38 years old)	Acute gout	Wrist, finger	540	84	None	24	60

### Case 1

A 72-year-old woman with a 13-year history of chronic tophaceous gout and hyperuricemia was treated with rasburicase. The patient previously had a severe cutaneous reaction to allopurinol, and uricosuric treatment with benzbromazone caused renal stones. During previous gout flares, medical treatment was unsatisfactory. She could only tolerate a low dose of diclofenac (50–100 mg/day), as higher doses caused gastrointestinal side-effects including one episode of gastrointestinal hemorrhage. Colchicine at 1 mg/day provoked intolerable diarrhea, and oral corticosteroid caused severe abdominal pain.

Uricase treatment was commenced (14 mg intravenously daily for 5 days) and resulted in rapid lowering of the patient's uric acid levels. On the fourth day of treatment, arthritis developed in her hand and foot joints. As treatment with diclofenac during previous flares took more than 1 week to relieve her symptoms, treatment with anakinra was started. Her arthritis responded rapidly and she was able to continue the course of uricase. No acute flares were observed during the following 2 months.

### Case 2

A 70-year-old man with an 8-year history of chronic tophaceous gout was assessed for hypouricemic treatment. The patient's past medical history included congestive cardiac failure, severe ischemic heart disease, hypertension and renal insufficiency (serum creatinine, 202 μmol/l; normal range, 44–80 μmol/l). Previous trials of treatment with allopurinol had to be abandoned because acute gout developed after the first dose, which did not respond to small doses of NSAIDs. Higher doses of NSAIDs were contraindicated because of renal failure. Colchicine at low doses (<1 mg/day) provoked rapid onset of diarrhea.

The patient was again started on a low dose of allopurinol (100 mg), and after the first dose developed acute arthritis of the right foot and ankle. Anakinra was administered for 3 days with rapid and complete resolution of signs and symptoms of arthritis. The patient continued on allopurinol 100 mg daily, and at follow-up 2 months later he had no further flare-ups while continuing on the same dose of allopurinol.

### Case 3

A 72-year-old man with a past history of diabetes, hypertension, renal failure and ischemic heart disease presented with polyarticular gout. Arthritis involved the knees, the right ankle and the right tarsal joints. MSU crystals were detected in the knee joint aspirate.

Owing to renal impairment and a history of rectal bleeding on colchicine, treatment with oral prednisone at 30 mg daily was started with a tapering dose over 7 days. Despite steroids, the arthritis remained active and the patient could not walk. After steroids were stopped, the patient received a 3-day course of anakinra, with complete resolution of arthritis by the second day. There had been no recurrence of arthritis at follow-up 1 month later.

### Case 4

A 50-year-old man with a 20-year history of polyarticular gout principally involving the right ankle and big toe sought medical advice because of increasingly frequent gout attacks. He had started allopurinol but could not continue treatment because it induced flare-ups of arthritis. NSAIDs caused severe gastrointestinal pain, and colchicine at 1 mg daily was ineffective. Higher doses of colchicine provoked diarrhea. On examination, he had arthritis of the right ankle joint and MSU crystals were identified in the joint aspirate.

A trial of anakinra was initiated at the same time as starting allopurinol (300 mg/day). The patient's arthritis responded rapidly and he continued on allopurinol. He was asymptomatic 2 months later.

### Case 5

A 40-year-old man with a 12-year history of gout presented with recurrent podagra of the right big toe. The attacks occurred approximately once every 2 months, and responded normally to indomethacin 150 mg daily over 7 days. The patient was taking allopurinol 300 mg daily and his uric acid level was stable at 322 μmol/l. He could not tolerate colchicine, which induced diarrhea when given at doses above 1.5 mg daily.

The patient consulted after another attack, which did not respond to indomethacin over 2 days. Joint aspiration confirmed the presence of urate crystals in the joint fluid. The patient received anakinra and his symptoms completely resolved 36 hours after the first injection. He has had no further attacks at the 6-week follow-up.

### Case 6

A 73-year-old female presented with acute gout involving the big toes of both feet for the first time. She had a past medical history of hypertension and knee osteoarthritis. On examination, both metatarsophalangeal-1 joints were red and swollen and tender to the touch. The joint aspirate did not yield any fluid. Serum uric acid was raised at 572 μmol/l and serum creatinine was normal at 72 μmol/l. A presumptive diagnosis of acute gout was made because of the patient's clinical presentation and the hyperuricemia.

The patient's symptoms had not responded to 2 days of treatment with diclofenac (100 mg daily), so anakinra was started. Her joint pains were 50% improved by 24 hours and 80% by 36 hours after the injections. No further recurrence was reported on follow-up at 1 month. The patient was started subsequently on allopurinol.

### Case 7

A 76-year-old man developed polyarticular gout affecting his right ankle, the right tarsal joint and both big toe joints during hospitalization for acute cholecystitis and bacterial sepsis. The patient had a past medical history of cardiovascular disease treated with low-dose aspirin and nitrates, of hypertension, of obstructive airway disease and of cirrhosis. On examination, joint inflammation was observed at the aforementioned affected sites. Aspiration of the ankle joint revealed uric acid crystals.

Owing to a lack of response to NSAIDs over 48 hours, anakinra treatment was started. The patient responded after the first injection, and the symptoms and signs of gout were completely abolished 48 hours afterwards.

### Case 8

A 70-year-old man developed arthritis of his right wrist, elbow, hand and shoulder. The patient's past medical history included chronic renal failure, diabetes, and cardiovascular disease with atrial fibrillation. Gout was suspected because of hyperuricemia and he was treated with colchicine, which provoked rectal bleeding and a worsening of renal failure. The patient's serum urate was raised at 779 μmol/l and his serum creatinine was 258 μmol/l. The joint aspiration of the left elbow revealed MSU crystals.

A trial of treatment with prednisone caused a decompensation of the patient's diabetes. Anakinra was started and on review, after completing the injections, the symptoms of arthritis of the knee and elbow were greatly reduced, but the right wrist and hand remained moderately painful.

### Case 9

A 53-year-old male presented with severe tophaceous gout of 17 years' duration. In the past, treatment with allopurinol and uricosurics had been attempted but discontinued because treatment provoked acute attacks of gout that were not well controlled by NSAIDs. Colchicine caused severe diarrhea at a dose of 1 mg/day. There was a positive family history of gout. On examination, tophi were detected over the elbows, fingers, knees, ankles and feet, with signs of inflammation in the hands, knees and right foot. The patient's serum urate was increased at 660 μmol/l and his renal function was normal.

Owing to the patient's prior intolerance of NSAIDs and colchicine, a further attempt to start allopurinol was made under cover of anakinra, given at 100 mg daily over 3 days. On starting anakinra, the patient reported a 50% decrease in joint pains after 2 days and he started allopurinol 200 mg daily and acetametacin 90 mg daily without complications. On follow-up 1 month later, the patient had one minor attack of gout and the allopurinol was continued.

### Case 10

A 38-year-old male presented with painful arthritis of the left wrist. In the past, the left wrist was fractured in a road traffic accident and the patient also had a past history of gout affecting the ankles and the left knee. Aspiration of the left wrist showed MSU crystals. Colchicine (2 mg/day) over 5 days was not effective in controlling the symptoms of gout.

Anakinra was administered and there was a rapid clinical response. On follow-up at day 7 after the first injection, the left wrist was still slightly swollen and tender but the ankle and knee were asymptomatic. The patient was subsequently started on allopurinol.

## Discussion

MSU crystals induce tissue inflammation via multiple mechanisms. The release of monocyte-derived cytokines, triggered by urate crystals, can in turn modulate endothelial expression of adhesion molecules that enhance neutrophil recruitment to the site of inflammation [16]. Within the joint, the release of chemokines, prostanoids as well as kinins further amplifies the inflammatory response [17]. The recent finding that MSU crystals induce IL-1 release by activation of the NALP3 inflammasome led us to investigate whether IL-1 blockade could inhibit inflammation provoked by MSU. Our results from animal studies confirmed the effectiveness of IL-1 inhibition in preventing neutrophil trafficking to the peritoneum, whereas TNF inhibition did not have any effect. Indeed, TNF inhibition aggravated neutrophil influx. At present we do not have any simple explanation for the apparent increase in neutrophil accumulation in mice treated with the anti-TNF mAb. This observation was made in the two separate experiments performed with five animals per group. The anti-TNF agent employed inhibited concavalin A-induced hepatitis, demonstrating that the reagent is biologically active.

The latter results differed from those reported by Chapman and colleagues, who found that anti-TNF inhibited MSU-induced endothelial activation, as evidenced by scintigraphy, in a porcine monoarthritis model [16]. These differences may be due to the different species employed in the animal models as well as to the differences in the cytokine inhibitors tested. Apart from the inflammasome, IL-1 secretion by macrophages in contact with MSU can also be induced through a TLR-dependent mechanism [5]. In the current experiments, IL-1 inhibitors would block the effects of activation via both the TLR and the inflammasome pathways.

As proof of concept that IL-1 inhibition may be clinically effective, we performed an open-label study of anakinra treatment in patients with acute gout. All patients had acute excacerbations of longstanding gout and three had severe tophaceous gout. All the patients had either not responded adequately to standard treatment with NSAIDs, colchicine or steroids (*n *= 7) or had significant comorbidities that made use of these treatments hazardous (*n *= 4). Three patients could not continue hypouricemic treatment with allopurinol because of the acute flares provoked at the start of treatment. Only three daily injections were administered, and in all cases the symptoms of gout responded rapidly. All patients had responded within 48 hours, and in four patients (cases 2, 3, 4 and 10) the symptoms improved within 24 hours. Patients' subjective assessment of pain due to gout was positive, and the mean diminution of pain was 79% by day 3 after the first injection. Not all patients responded so well, particularly in two of the patients who had tophaceous gout. They had a moderate response, although still with >50% reduction of pain. As IL-1 blockade may increase the risk of infection, we excluded any patient who had proven or clinically suspected active infection. When appropriate, joint aspiration was performed to exclude septic arthritis. No treatment-related side-effects were observed during therapy and there were no infectious complications. On follow-up, three patients who had tophaceous gout reported mild joint pains but no acute flare during the first month.

Although these findings are positive, we have to stress that this was an open-label study and confirmation of these results would require a randomized controlled trial to prove that IL-1 inhibition is effective in acute gout. If the effectiveness of IL-1 is confirmed, then a short course of IL-1 inhibition may prove to be a valid addition to the therapeutic arsenal when the physician is confronted with complicated cases of gouty arthritis.

## Conclusion

In this pilot study involving 10 patients with gouty arthritis refractory to conventional therapies, anakinra given at 100 mg daily for 3 days rapidly relieved the inflammatory symptoms of gout. These results reinforce the findings that implicate IL-1β in the pathophysiology of gout and need to be confirmed by randomized controlled trials. IL-1 inhibition may be a promising therapeutic target in crystal-induced arthritis.

## Abbreviations

IL = interleukin; mAb = monoclonal antibody; MSU = monosodium urate; NALP = Nacht, LRR and Pyrin domain containing protein; NSAID = nonsteroidal antiinflammatory drug; PBS = phosphate-buffered saline; TLR = Toll-like receptor; TNF = tumor necrosis factor.

## Competing interests

The authors declare that they have no competing interests.

## Authors' contributions

The study was conceived by AS and JT. TDS performed the animal studies. AS and SR performed the clinical study.

## References

[B1] Saag KG, Choi H (2006). Epidemiology, risk factors, and lifestyle modifications for gout. Arthritis Res Ther.

[B2] di Giovine FS, Malawista SE, Thornton E, Duff GW (1991). Urate crystals stimulate production of tumor necrosis factor alpha from human blood monocytes and synovial cells. Cytokine mRNA and protein kinetics, and cellular distribution. J Clin Invest.

[B3] Malawista SE, Duff GW, Atkins E, Cheung HS, McCarty DJ (1985). Crystal-induced endogenous pyrogen production. A further look at gouty inflammation. Arthritis Rheum.

[B4] Di Giovine FS, Malawista SE, Nuki G, Duff GW (1987). Interleukin 1 (IL 1) as a mediator of crystal arthritis. Stimulation of T cell and synovial fibroblast mitogenesis by urate crystal-induced IL 1. J Immunol.

[B5] Liu-Bryan R, Scott P, Sydlaske A, Rose DM, Terkeltaub R (2005). Innate immunity conferred by Toll-like receptors 2 and 4 and myeloid differentiation factor 88 expression is pivotal to monosodium urate monohydrate crystal-induced inflammation. Arthritis Rheum.

[B6] Martinon F, Petrilli V, Mayor A, Tardivel A, Tschopp J (2006). Gout-associated uric acid crystals activate the NALP3 inflammasome. Nature.

[B7] Martinon F, Tschopp J (2004). Inflammatory caspases: linking an intracellular innate immune system to autoinflammatory diseases. Cell.

[B8] Martinon F, Tschopp J (2005). NLRs join TLRs as innate sensors of pathogens. Trends Immunol.

[B9] Kanneganti TD, Ozoren N, Body-Malapel M, Amer A, Park JH, Franchi L, Whitfield J, Barchet W, Colonna M, Vandenabeele P (2006). Bacterial RNA and small antiviral compounds activate caspase-1 through cryopyrin/Nalp3. Nature.

[B10] Mariathasan S, Weiss DS, Newton K, McBride J, O'Rourke K, Roose-Girma M, Lee WP, Weinrauch Y, Monack DM, Dixit VM (2006). Cryopyrin activates the inflammasome in response to toxins and ATP. Nature.

[B11] Agostini L, Martinon F, Burns K, McDermott MF, Hawkins PN, Tschopp J (2004). NALP3 forms an IL-1beta-processing inflammasome with increased activity in Muckle–Wells autoinflammatory disorder. Immunity.

[B12] Hawkins PN, Lachmann HJ, Aganna E, McDermott MF (2004). Spectrum of clinical features in Muckle–Wells syndrome and response to anakinra. Arthritis Rheum.

[B13] Goldbach-Mansky R, Dailey NJ, Canna SW, Gelabert A, Jones J, Rubin BI, Kim HJ, Brewer C, Zalewski C, Wiggs E (2006). Neonatal-onset multisystem inflammatory disease responsive to interleukin-1beta inhibition. N Engl J Med.

[B14] Ortiz-Bravo E, Schumacher HR (1993). Components generated locally as well as serum alter the phlogistic effect of monosodium urate crystals *in vivo*. J Rheumatol.

[B15] Wallace SL, Robinson H, Masi AT, Decker JL, McCarty DJ, Yu TF (1977). Preliminary criteria for the classification of the acute arthritis of primary gout. Arthritis Rheum.

[B16] Chapman PT, Yarwood H, Harrison AA, Stocker CJ, Jamar F, Gundel RH, Peters AM, Haskard DO (1997). Endothelial activation in monosodium urate monohydrate crystal-induced inflammation: *in vitro *and *in vivo *studies on the roles of tumor necrosis factor alpha and interleukin-1. Arthritis Rheum.

[B17] Terkeltaub R, Zachariae C, Santoro D, Martin J, Peveri P, Matsushima K (1991). Monocyte-derived neutrophil chemotactic factor/interleukin-8 is a potential mediator of crystal-induced inflammation. Arthritis Rheum.

